# In Vitro Evaluation of *Leuconostoc mesenteroides* Cell-Free-Supernatant GBUT-21 against SARS-CoV-2

**DOI:** 10.3390/vaccines10101581

**Published:** 2022-09-21

**Authors:** Othman R. Alzahrani, Yousef M. Hawsawi, Abdullah D. Alanazi, Hanan E. Alatwi, Irfan A. Rather

**Affiliations:** 1 Department of Biology, Faculty of Science, University of Tabuk, Tabuk 71491, Saudi Arabia; 2 Genome and Biotechnology Unit, Faculty of Science, University of Tabuk, Tabuk 71491, Saudi Arabia; 3 Research Center, King Faisal Specialist Hospital and Research Center, Jeddah 21499, Saudi Arabia; 4 College of Medicine, Al-Faisal University, P.O. Box 50927, Riyadh 11533, Saudi Arabia; 5 Department of Biological Sciences, Faculty of Science and Humanities, Shaqra University, P.O. Box 1040, Ad-Dawadimi 11911, Saudi Arabia; 6 Department of Biological Sciences, Faculty of Science, King Abdulaziz University, Jeddah 21589, Saudi Arabia; 7 Center of Biological Sciences, Faculty of Science, King Abdulaziz University, Jeddah 21589, Saudi Arabia; 8 Department of Applied Microbiology and Biotechnology, Yeungnam University, Gyeongsan 38541, Gyeongbuk, Korea

**Keywords:** SARS-CoV-2, *Leuconostoc mesenteroides*, probiotics, virus, lactic acid bacteria

## Abstract

The unprecedented health catastrophe derived from the severe acute respiratory syndrome coronavirus-2 (SARS-CoV-2 infection) met with a phenomenal scientific response across the globe. Worldwide, the scientific community was focused on finding a cure for the deadly disease. A wide range of research studies has consistently revealed the link between SARS-CoV-2 infection severity and abnormal gut microbiomes, suggesting its potential in developing novel therapeutic approaches. Probiotics have been extensively studied to promote health in human hosts and reestablish a balance in the dysbiotic gut microbiome; however, there is strong skepticism about their safety and efficacy. Consequently, the metabolic signatures of probiotics, often referred to as "postbiotics", could prove of paramount importance for adjuvant cures in patients with SARS-CoV-2. Postbiotics exhibit safety, enhanced shelf-life, and stability and, therefore, could be implemented in SARS-CoV-2 prophylactic strategies with no undue adverse side effects. The current study is a preliminary investigation of the antiviral properties of postbiotic metabolites derived from *Leuconostoc mesenteroides* GBUT-21. The study focuses on the potential biological role in inactivating SARS-CoV-2 and reducing related inflammatory pathways.

## 1. Introduction

COVID-19 escalated into a global pandemic in 2020, caused by a novel, highly contagious human coronavirus variant called severe acute respiratory syndrome coronavirus 2 (SARS-CoV-2). In addition to COVID-19, two zoonotic coronaviruses have spread from animal reservoirs to humans in the past two decades, leading to epidemic outbreaks of severe acute respiratory syndrome coronavirus (SARS-CoV) across 2002–2004, along with ongoing intermittent episodes of Middle East respiratory syndrome coronavirus (MERS- CoV) [1,2,3,4]. The SARS-CoV-2 virus is an enveloped, positive-sense, single-stranded RNA virus that can replicate in the lower human respiratory tract [5]. The virus transmits in human hosts through aerosols and respiratory droplets with an incubation period of 4–5 days before the onset of disease symptoms [6,7,8]. The severity of infection may vary from asymptomatic (with or without detectable virus) to mild or moderately symptomatic with the presence of detectable virus, experiencing nonspecific clinical manifestations such as fever, chills, sore throat, muscle pain, cough, headache, diarrhea, and loss of smell and taste [8,9,10,11,12]. The severe SARS-CoV-2 pathology is marked by the presence of heavy viral load and the onset of the disease called dyspnoea or shortness of breath due to hypoxemia, which could progress to a potentially fatal condition, acute respiratory distress syndrome (ARDS), or closely related pneumonia [12,13,14,15]. Research suggests that most patients with COVID-19 are either asymptomatic or with mild or moderate infection but approximately 10% experience a severe or potentially lethal illness [16]. The COVID-19 vaccine program continues at the frontier of immune defense against COVID-19 infection worldwide. However, effective treatment options need to be explored, especially for underdeveloped countries where procurement and implementation of vaccine regimens are slow and challenging. In addition, the constant mutations in SARS-CoV-2 RNA potentially make it unrecognized by the existing vaccines. 

Postbiotics are active substances produced by microorganisms during their metabolic activities and are known to exhibit a promising impact on host health. Postbiotics are a thrust area of research nowadays, and there is a growing continuum of studies highlighting their potential in combating viral infections, including SARS-CoV-2 infection. Biologically potent postbiotic metabolites are accessed through the filtered culture of cell-free supernatants from a bacterial culture. A study on *Lactobacillus acidophilus* and *Lactobacillus casei* supernatants showed anti-inflammatory and antioxidant effects on intestinal epithelial cells, resident macrophages, and neutrophils. The cell-free supernatant reduced the levels of the proinflammatory tumor necrosis factor α (TNF-α) cytokine and increased secretion of the anti-inflammatory cytokine interleukin 10 (IL-10) [17]. Therefore, postbiotics could decrease the intensity of SARS-CoV-2 via their anti-inflammatory efficacy. A recent study on supernatants derived from *Lactobacillus* and *Bifidobacterium* cultures showed that they effectively prevent the invasion of enteroinvasive *E. coli* strains into enterocytes in vitro [18]. Postbiotics may reduce gut inflammation via the activation of regulatory T cells and reduce systemic inflammation by fortifying the intestinal barrier to inhibit the translocation of the virus to extra-intestinal organs. One such study showed that the metabolically active supernatants of *Lactobacillus plantarum* were beneficial for the intestinal barrier’s maturation and morphological structure [19]. The supernatant also decreased the inflammatory marker concentrations (IL-1β and TNF-α) in the intestinal mucosa [19]. In a randomized clinical trial, subjects fed with probiotic *Lactobacillus rhamnosus* GG were less likely to develop COVID-19 symptoms compared to placebo [20,21,22,23,24,25,26,27,28,29]. A bidirectional gut-lung microbiome axis has also been uncovered recently, which explains how microbiomes across the lung and gut cross-communicate and, therefore, influence the host’s immune health [30,31,32,33,34,35]. Nutraceuticals, such as probiotics, contain lactic acid bacteria and have been used in food for centuries to enhance immunity and fight infection. In a randomized, double-blind, placebo-controlled trial, probiotic administration for seven days induced the anti-inflammatory cytokine production (IL-10 and TGF-β1) while decreasing the pro-inflammatory (IL-6, IL-12p70, IL-17, and TNF-α) cytokines involving 100 children with severe sepsis [36]. Further support for this view was provided by a recent meta-analysis showing that probiotic treatment reduced IL-6 and C-reactive protein levels among middle-aged and older individuals with chronic low-grade inflammation [37]. Despite their outstanding credentials, some studies have shown adverse clinical and technological effects of probiotics, such as limited strain survivability and lifespan, presence of virulence factors in certain strains, unique colonization patterns specific to the strain, opportunistic infection-causing ability in immunocompromised individuals, production of biogenic amines, unclear dosage instructions across the globe, etc [38]. Thus, functional bioactive molecules released by probiotics termed postbiotics have become thrust research areas in recent years [39,40]. 

Despite the intricate interconnection between invasive viruses, the gastrointestinal microbiome, and host physiology, scientists are endeavoring to establish new ways to combat COVID-19, including the use of postbiotics as potential prophylactic. This study evaluates the efficacy of postbiotics produced by *Leuconostoc mesenteroides*-enriched cell-free supernatant GBUT-21, extracted from fermented camel milk in viral replication and immune modulation.

## 2. Materials and Methods

### 2.1. Isolation and Bacteria Growth

The fermented camel milk was lightly homogenized for isolating lactic acid bacteria and then serially diluted from 10^−1^ to 10^−6^ using sterile phosphate buffer [41,42]. A 100 µL tube from each dilution was plated on BCP agar plates and incubated at 37 °C for 24 h to see the clear zone around the colonies. The clear zones around colonies were considered to be the presence of lactic acid bacteria. Each colony was carefully picked with a sterile loop and inoculated in MRS broth. The inoculated MRS broth was incubated at 37 °C for 24 h. Then, the samples were spread on MRS agar and preserved at −80 °C for further use. Colonies that did not show a clear zone on BCP agar were discarded.

### 2.2. Bacteria Sample Preparation and Screening against SARS-CoV-2

The selected LAB isolates were grown following the same procedure as stated above. The culture was centrifuged at 5000 × g for 10 min, and the supernatant was collected and filter-sterilized using a syringe microfilter. The collected cell-free supernatant was freeze dried in sterilized condition and stored at −80 °C until used. The selected isolates were screened by culturing SARS-CoV-2 in HEK cells at approximately 80–90% confluency for 2 days in a BSL-3 environment [43]. HEK cells infected with the virus were treated with different concentrations of samples. Only bacteria strains showing potent inhibition of the virus were selected for further characterization.

To further check the in vitro antiviral inhibition of GBUT-21, we followed the procedure described by Rather et al. [43]. Briefly, the human embryonic kidney epithelial cells (HEK-293 cells) with a cell density of approximately 4 × 10^4^ per well were seeded in 96 well plates. After 24 h of incubation, the cells were incubated with 100 μL culture medium for virus infection (DMEM supplemented with penicillin (100 U/mL) and streptomycin (100 μg/mL) + GBUT-21-CFS (20, 40, 60 mg/mL *w*/*v*) + SARS-COV-2 virus (0.1 multiplicity of infection)). The cells were washed three times with PBS after 2 h of incubation at 37 °C and then maintained in 100 μL of DMEM without virus. In addition, a separate virus control was also inoculated without GBUT-21. Inhibition of virus load was quantified using rt-PCR.

### 2.3. Biochemical Characterization of LAB Isolate 

During the screening of LAB isolates against SARS-CoV2, *Leuconostoc mesenteroides*-enriched supernatant, labeled as GBUT-21, showed strong viral inhibition. The biochemical characterization of the strain was carried out using API 50CH strips supplied with API 50CHL medium. The protocol was followed as per the instructions provided by the manufacturer [41,44,45,46]. Additionally, the molecular characterization of GBUT-21 was conducted via 16S rRNA gene sequencing at Microgen, South Korea. The sequence received was registered in GenBank for an accession number.

### 2.4. Determination of Cytotoxity 

Following the protocol depicted elsewhere [43], Hela, HCE, and HEK-293 cells were cultured in DMEM, supplemented with 10% FBS, penicillin 100 U/mL, and streptomycin 100 µg/mL. Then, the cells were incubated in a cell culture incubator supplied with 5% CO_2_ maintained at 37 °C. After 24 h of incubation, the plates were checked for contamination, and cells with no contamination were treated with different concentrations of GBUT-21 prepared in DMEM. Similarly, an intact control cell was treated with only PBS and DMEM.

### 2.5. MTT Assay 

After the incubation, as stated in Section 2.5 above, the cells in each well were washed with PBS, and then, each well was treated with 5 mg/mL of MTT solution. The plates were incubated at 37 °C for 45 to 50 min. The supernatant for each well was carefully discarded, followed by dissolving formazan crystals in isopropanol. Finally, the absorbance was measured at 570 nm.

### 2.6. Estimation of Intracellular ROS 

The ROS was measured from each group at 70% cell confluence. Briefly, when the cells reached 70% confluence, they were treated with 10 µM of DCFH-DA prepared in DMEM [43]. The plates were incubated at 37 °C for 30 min. Finally, each well was washed with PBS, and the intensity of fluorescence was checked using a fluorescence microscope.

### 2.7. Immunofluorescent Assay

For immunocytochemistry, the HEK 293 cells from each group were washed prior to fixing them in 4% formaldehyde for 15 min. Subsequently, cells were permeabilized with Triton X (0.2%), followed by a signal enhancer [43]. Then, cells were blocked with five percent PBS diluted goat serum and incubated with a primary antibody of pERK (1:100) at 4 °C for 15 h. Then, the cells were incubated with secondary antibodies after washing 2–3 times with sterile PBS for 50–60 min. Finally, cell nuclei were counterstained using DAPI, followed by PBS wash after rinsing with PBS. The images were taken by a camera attached to a fluorescent microscope.

### 2.8. RT-qPCR

The RNA extraction was performed using a Quick-RNA Viral Kit following the manufacturer’s protocol. The RNA was eluted in nuclease-free water and then 1 μg of RNA with a total volume of 25 µL in a reaction mixture primed with random primers. The combination was reverse transcribed for 5 min at 73 °C and 60 min for 37 °C, following the same steps described elsewhere [43]. Real-time PCR was performed with 2 μL of cDNA, 10 pmol of each gene-specific primer, and Power SYBR® Green PCR Master Mix on a 7500 real-time PCR system.

## 3. Results

### 3.1. Selection and Characterization of Lactic Acid Bacteria Isolate

To identify an LAB isolate, a colony that shows a yellow zone on the BCP agar plate was confirmed as the presence of the lactic acid-producing bacterium. The selected colonies were carefully picked and inoculated in MRS broth. Here, a total of 23 LAB were isolated from fermented camel milk. However, only one strain, GBUT-21, showed strong antiviral activity against SARS-CoV-2.

The biochemical characterization of GBUT-21 was carried out using an API50 KIT, where the strain was seen close to *Leuconostoc mesenteroides*. A total of 22 carbohydrates showed complete fermentation that changed color from violet in the strop capsule to yellow. However, one carbohydrate was seen partially fermented in all three triplicate experiments, which could be the result of less amount of culture inoculum. Overall, we considered the partially fermented carbohydrate as fermented. Therefore, 23 carbohydrates showed fermentation, as shown in Table S1. 

The strain was further identified via molecular characterization using 16S rRNA gene sequencing and was identified as *Leuconostoc mesenteroides*, and its supernatant hereafter named *Leuconostoc mesenteroides* GBUT-21. The sequence was registered in GenBank with accession number ON616405.

### 3.2. Virus Inhibition and Cell Viability

**Figure 1:**
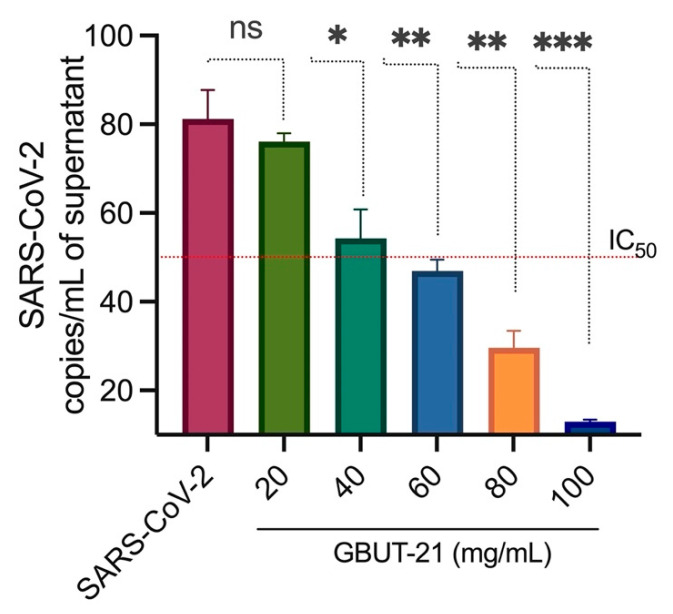
Inhibition of late-stage viral infection in HEK293. All experiments were performed in triplicate. The results are presented as average values with standard deviations. An asterisk (*p* < 0.05) indicates values that are significantly different from the control. Bars depict mean ± SD of three independent experiments. *** *p* < 0.001, ** *p* < 0.01, * *p* < 0.05 vs. virus (infected).

**Figure 2:**
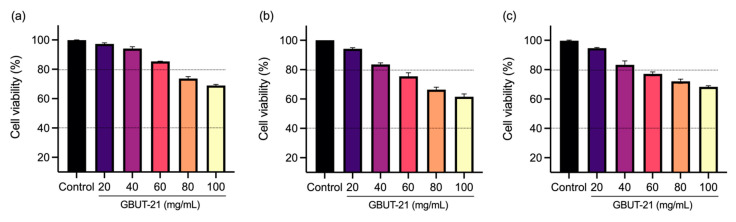
The therapeutic concentration of GBUT11 CSF is well-tolerated by human cell lines. MTT assay was performed on HeLa, HEK, and HCE cell lines to check the percentage viability in the presence of the sample. Cells were seeded in 96-well plates at a density of 4 × 10^5^ and treated with the sample for 24 h. Percentage viability: (**a**) HEK, (**b**) HeLa, and (**c**) HCE cells at different concentrations of the sample.

The bioactive compounds produced using probiotics hold curative potential against a number of diseases. Here, we first evaluated the antiviral effect of a *Camelus* isolates *Leuconostoc mesenteroides* GBUT-21 cell-free supernatant in the SARS-COV-2 virus-infected HEK293 cells. The cells were treated with different concentrations of the GBUT-21, as shown in Figure 1. Quantitative PCR (qPCR) analysis revealed a reduction in viral copies in the culture supernatant treated with an increasing concentration of GBUT-21 CSF compared to the DMSO-treated control sample (Figure 1). MTT analysis excluded any possibility of cytotoxicity in mammalian cells, including HEK293, HCE, and Hela. GBUT-21 CSF tolerance was highest in HEK293, followed by HCE cells and Hela (Figure 2). Above 80% of cell viability was maintained in Hek293 and HCE cells when treated with 40–60 mg/mL of GBUT-21 CSF. However, cells showed sensitivity to the cell supernatant beyond 80 mg/mL GBUT-21 CSF as the concentration resulted in less than 80% cell viability (Figure 2). The results confer that GBUT-21 CSF could inhibit the replication of contagious human coronavirus without any profound cytotoxicity effect if administered at 40–60 mg/mL of concentration; therefore, the dosage at 40–60 mg/mL was used for all subsequent experiments.

### 3.3. Estimation of Intracellular ROS

**Figure 3:**
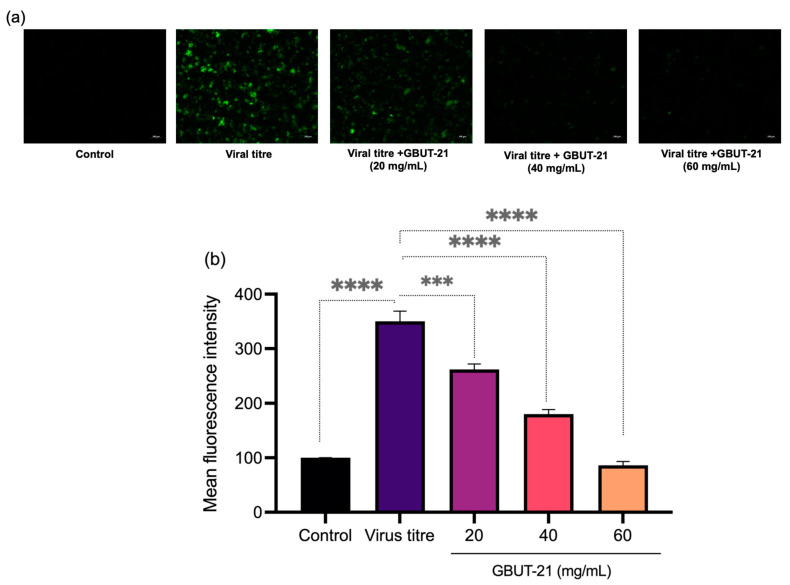
ROS staining via H2DCFDA staining. Treatment cells were stained with H2DCFDA stain for 30 min and PBS washes were further given to get final fluorescence images under fluorescence microscope. (**a**) Fluorescence images. (**b**) Depicting mean fluorescence intensity. An asterisk (*p* < 0.05) indicates values that are significantly different from the control. Bars depict mean ± SD of three independent experiments. **** *p* < 0.0001, *** *p* < 0.001 vs. virus (infected).

Several studies have linked the cytokine storm to the deterioration of the patient’s condition when suffering from COVID-19 infection. It plays a significant role in developing acute respiratory distress syndrome (ARDS) and multiple organ dysfunction [10,11,47,48,49,50]. Cytokine storms occur when there is an unregulated host immune response to different triggers resulting in the auto-amplifying production of cytokines. The cytokine storm cycle is perpetuated by oxidative stress due to hypoxia or increased intracellular reactive oxygen species (ROS) levels in COVID-19 patients. Therefore, we continued examining the effect of GBUT-21 CSF on the intracellular ROS levels produced in Hek293 cells infected with the SARS-CoV-2 virus. Corroborating the previous studies, we found a significant increase in ROS levels by approximately 3.5-fold after the infection of SARS-COV-2. Interestingly, when the infected cells were treated with 20, 40, and 60 mg/mL of GBUT-21 CSF, ROS levels significantly reduced to 2.5, 1.7-fold, and 0.9-fold, respectively (Figure 3).

### 3.4. Antiviral Effect Depicted by Immunofluorescent Assay

**Figure 4:**
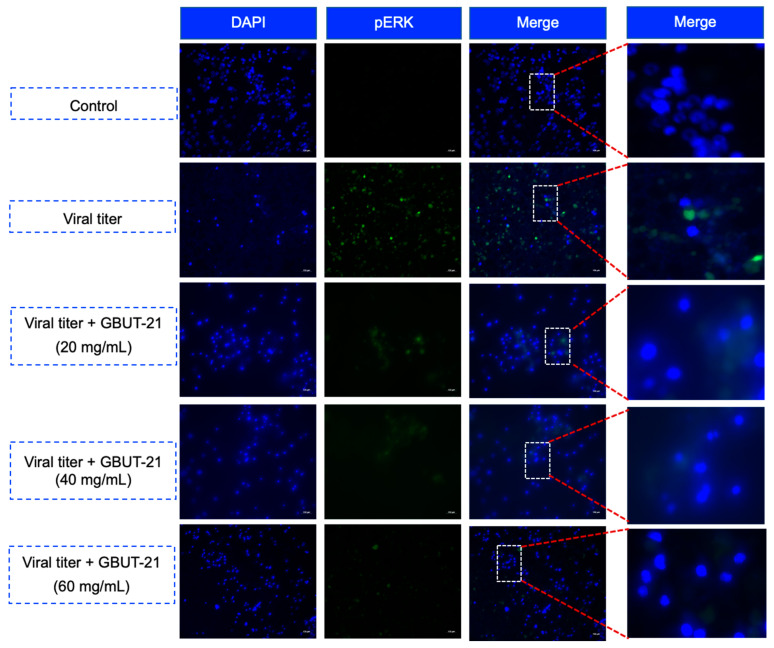
The influence of GBUT-21 on p-ERK protein in HEK293 cells after infection with SARS-CoV-2.

Activating intracellular signaling pathways, such as mitogen-activated protein kinase (MAPK), are crucial for producing cytokines during a SARS-CoV-2 infection [51]. The increasing proinflammatory cytokine production through these pathways damages airway epithelial cells and alveolar tissues and plays a central role in establishing a cytokine storm [52,53,54]. Viral activation of the MAPK pathway results in the phosphorylation of ERK protein (p-ERK) and is often associated with viral infections. In mock non-infected HEK293 cells treated with 0.1% DMSO, the expression of p-ERK was virtually non-existent, whereas in SARS CoV-2-infected cells treated with 0.1% DMSO, the expression of p-ERK was at its peak across the nucleus and cytoplasm. Interestingly, an increasing concentration of GBUT-21 CSF (20 mg/mL, 40 mg/mL, and 60 mg/mL) drastically reduced the phosphorylation of ERK in SARS-CoV-2-infected cells, as shown in Figure 4.

### 3.5. Gene Expression Profile

**Figure 5:**
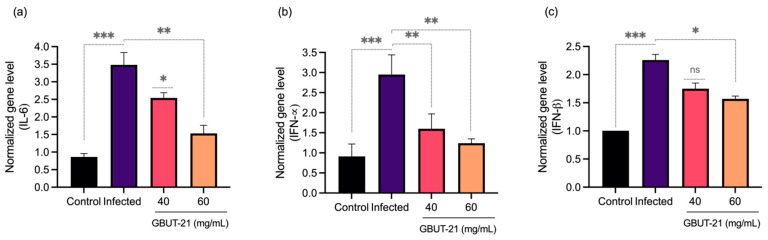
Gene expression of inflammatory markers in HEK 293 cells post infection with and without treatment of GBUT-21. (**a**) IL-6. (**b**) IFN-α. (**c**) IFN-β. An asterisk (*p* < 0.05) indicates values that are significantly different from the control. Bars depict mean ± SD of three independent experiments. *** *p* < 0.001, ** *p* < 0.01, * *p* < 0.05 vs. virus (infected).

Hyperinflammation in patients infected with SARS-CoV-2 is marked by elevated serum levels of proinflammatory cytokines such as IL-6, IFN-α, and IFN-β [55]. Patients with COVID-19 have shown deteriorating lung function is almost exclusively related to the inflammatory cytokine IL-6 [56]. We observed an upregulation of the proinflammatory cytokine genes such as IFN-α, IFN-β, and IL-6 in SARS-CoV-2 infected cells compared to the non-infected mock cells (Figure 5). Interestingly, GBUT-21 CSF treatment resulted in a two-fold reduction in the expression of IFN- α and IL-6 genes, respectively. However, IFN-β did not show a stark decrease in expression upon GBUT-21 treatment in SARS-CoV-2-infected cells. The findings suggest that GBUT-21 CSF exhibits antiviral activity and regulates the inflammatory responses to the virus invasion.

## 4. Discussion

In this study, we investigated the antiviral activity of metabolically active cell-free supernatant against SARS-CoV-2 from the culture of *Leuconostoc mesenteroides* GBUT-21. The active compounds in GBUT-21 CSF eradicated the virus to a large extent, with the effect becoming more pronounced with increased concentrations. The postbiotic present in the GBUT-21 CSF would have exerted an anti-SARS-CoV-2 effect through competitive inhibition by directly binding at the ligand-binding pockets of the ACE2 receptor [57]. Several studies have investigated the receptor blocking effect of bacteriocins, a *lactobacillus* postbiotic compound, and found that plantaricin forms strong hydrogen bonds with ACE2 receptors [57,58,59,60] and the residual binding domain (RBD) of spike protein S [57]. Therefore, bacteriocins present in the postbiotics could form a potent competitive inhibitor for the SARS-CoV-2 virus. In addition, an intracellular mechanism for the antiviral activity of postbiotics has been proposed, which suggests that the postbiotic metabolite may interfere in essential processes such as viral RNA replication. An in silico study by Anwar et al. demonstrated that bacteriocin was able to bind tightly by blocking RNA-dependent RNA polymerase (RdRp) of SARS-CoV-2, which inhibited the viral replication cycle in host cells [57]. A parallel in silico molecular docking study showed antiviral activity of a postbiotic derived from *L. plantarum* Probio-88 using plantaricins against SARS CoV-2 helicase nsp13 [43]. Helicases have the role of separating self-annealed ss-RNA by using ATP as an energy source. The study confirmed the establishment of hydrogen bonds and a high binding affinity of plantaricins to helicase nsp13, which might serve as a blocker by preventing the binding of ss-RNA on helicase. Therefore, it is highly likely that GBUT-21 CSF has bacteriocin activity, and the reduction in viral load could either be due to competitive inhibition at the ACE2 or at the residual binding domain (RBD) of spike protein S. The reduction in SARS-CoV-2 load could also be as a result of inhibition of RNA-dependent RNA polymerase (RdRp) or helicase nsp13. 

The treatment with GBUT-21 CSF in SARS-CoV-2-infected cells reduced the inflammatory cytokines (IL6, INF-alpha, and INF-beta), which is also corroborated by the decrease in the reactive oxygen species (ROS) levels and reduced activity of the Raf/MEK/ERK (p-ERK) signaling pathway. Researchers suggest that after the successful viral invasion, the activation of intracellular signaling pathways, such as mitogen-activated protein kinase (MAPK) and nuclear factor kappa B (NF-κB), is vital for producing cytokines [51]. Through these pathways, the increased production of pro-inflammatory cytokines, particularly IL-6, results in decreased ventilation, acute lung injury, and ARDS, culminating in a life-threatening cytokine storm [52,53,54]. Therefore, a reduction in the levels of pro-inflammatory cytokines, including IL-6, after the treatment with GBUT-21 CSF exhibits the protective role of postbiotics against the SARS-CoV-2 virus. The reduced activity of the Raf/MEK/ERK signaling pathway and cytokine levels after the treatment with GBUT-21 CSF may help combat the life-threatening cytokine storm, which is a significant cause of death due to SARS-CoV-2 infection.

The preliminary data depicted in this study substantially indicates that the metabolically active postbiotics derived from *Leuconostoc mesenteroides* GBUT-21 could be protective against SARS-CoV-2 infection. Nonetheless, it is too early to determine exactly how the *Leuconostoc mesenteroides* GBUT-21 works. Therefore, it is important to unravel molecular signaling details and validate clinical outcomes in the future. Additionally, the current work only involves cell line models; future validation of the strain in animal models would be highly beneficial.

## 5. Conclusions

Over the past two years, enormous strides have been carried out in understanding the molecular mechanism and bioactive metabolites in postbiotics that could be effective against viruses such as the deadly SARS-CoV-2. Probiotic effector molecules are being continuously refurbished as postbiotics, allowing them to provide health benefits that radically open basic research to translational aspects. However, the lack of comprehensive data on the antiviral effects of postbiotics underscores the need for further investigation. However, the existing evidence suggests that these molecules possess extraordinary properties from clinical, technological, and economic perspectives. We demonstrated that the postbiotic metabolites derived from *Leuconostoc mesenteroides* GBUT-21 exhibited SARS-CoV-2 inhibitory activity and could form part of precision postbiotics or be used in an adjunct therapy along with vaccines for effective therapeutic and preventive interventions against this deadly disease. In addition, the active metabolites in the cell-free supernatant demonstrated potent anti-inflammatory activities, which could help mitigate a life-threatening condition, the cytokine storm, in COVID-19 patients.

## Data Availability

The data generated in this study are cited in the manuscript.

## Supplementary Materials

The following supporting information can be downloaded at: https://www.mdpi.com/article/10.3390/vaccines10101581/s1, Table S1: Biochemical characterization of *Leuconostoc mesenteroides* GBUT-21.
