# Supplementary material for: In Vitro Evaluation of Leuconostoc mesenteroides Cell-Free-Supernatant GBUT-21 against SARS-CoV-2
Source: Vaccines (Basel). 2022 Sep 21;10(10):1581. doi: 10.3390/vaccines10101581 (PMC9612097; doi:10.3390/vaccines10101581)
Supplement: Supplementary file 1 [file vaccines-10-01581-s001.zip › vaccines-1831647-supplementary.pdf]

Table S1. Biochemical characterization of *Leuconostoc mesenteroides* GBUT-21

Active ingredients	Results	Active ingredients	Results
Glycerol	–	Salicin	–
Erythritol	–	D-Cellobiose	+
D-Arabinose	–	D-Maltose	+
L-Arabinose	+	D-Lactose (bovine origin)	+
D-Ribose	+	D-Melibiose	+
D-Xylose	+	D-Saccharose	+
L-Xylose	–	D-Trehalose	+
D-Adonitol	–	Inulin	–
Methyl-β-D-Xylopyranoside	–	D-Melezitose	–
D-Galactose	+	D-Raffinose	+
D-Glucose	+	Amidon (starch)	–
D-Fructose	+	Glycogen	–
D-Mannose	+	Xylitol	–
L-Sorbose	–	Gentiobiose	+
L-Rhamnose	–	D-Turanose	+
Dulcitol	+	D-Lyxose	–
Inositol	–	D-Tagatose	–
D-Mannitol	–	D-Fuccose	–
D-Sorbitol	+	L-Fuccose	–
Methyl-α-D-Mannopyranoside	–	D-Arabitol	–
Methyl-α-noside	–	L-Arabitol	–
N-acetylglucosamine	+	Potassium Gluconate	+
Amygdalin	+	Potassium 2-Ketogluconate	–
Arbutin	+	Potassium 5-Ketogluconate	–
Esculin	+		

(-) The bacterium does not use this carbohydrate. (+) The bacterium uses this carbohydrate.
